# Specific gene expression signatures of low grade meningiomas

**DOI:** 10.3389/fonc.2023.1126550

**Published:** 2023-03-01

**Authors:** Erdyni N. Tsitsikov, Sanaa Hameed, Sherwin A. Tavakol, Tressie M. Stephens, Alla V. Tsytsykova, Lori Garman, Wenya Linda Bi, Ian F. Dunn

**Affiliations:** ^1^ Department of Neurosurgery, University of Oklahoma Health Sciences Center, Oklahoma City, OK, United States; ^2^ Department of Microbiology and Immunology, University of Oklahoma Health Sciences Center, Oklahoma City, OK, United States; ^3^ Department of Neurosurgery, Brigham and Women’s Hospital, Harvard Medical School, Boston, MA, United States

**Keywords:** meningioma, CNS tumors, transcriptome profiling, gene expression, *NF2*, *TRAF7*, *AKT1*, *KLF4*

## Abstract

**Introduction:**

Meningiomas are the most common primary central nervous system (CNS) tumors in adults, representing approximately one-third of all primary adult CNS tumors. Although several recent publications have proposed alternative grading systems of meningiomas that incorporate genomic and/or epigenomic data to better predict meningioma recurrence and progression-free survival, our understanding of driving forces of meningioma development is still limited.

**Objective:**

To define gene expression signatures of the most common subtypes of meningiomas to better understand cellular processes and signaling pathways specific for each tumor genotype.

**Methods:**

We used RNA sequencing (RNA-seq) to determine whole transcriptome profiles of twenty meningiomas with genomic alterations including *NF2* inactivation, loss of chr1p, and missense mutations in *TRAF7*, *AKT1* and *KLF4*.

**Results:**

The analysis revealed that meningiomas with *NF2* gene inactivation expressed higher levels of *BCL2* and *GLI1* compared with tumors harboring *TRAF7* missense mutations. Moreover, NF2 meningiomas were subdivided into two distinct groups based on additional loss of chr1p. NF2 tumors with intact chr1p were characterized by the high expression of tumor suppressor *PTCH2* compared to NF2 tumors with chr1p loss. Taken together with the high expression of *BCL2* and *GLI1*, these results suggest that activation of Sonic Hedgehog pathway may contribute to NF2 meningioma development. In contrast, NF2 tumors with chr1p loss expressed high levels of transcription factor *FOXD3* and its antisense RNA *FOXD3-AS1*. Examination of TRAF7 tumors demonstrated that TRAF7 regulates a number of biomechanically responsive genes (*KRT6a*, *KRT16*, *IL1RL1*, and *AQP3* among others). Interestingly, AKT1 and KLF4 meningiomas expressed genes specific for PI3K/AKT signaling pathway, suggesting overlapping gene signatures between the two subtypes. In addition, KLF4 meningiomas had high expression of carcinoembryonic antigen family members *CEACAM6* and *CEACAM5*.

**Conclusions:**

Each group of meningiomas displayed a unique gene expression signature suggesting signaling pathways potentially implicated in tumorigenesis. These findings will improve our understanding of meningioma tumorigenesis and prognosis.

## 1 Introduction

Meningiomas, named for their cell of origin, are the most common intracranial tumors in adults, representing 39% of all primary adult central nervous system (CNS) tumors (1). The World Health Organization (WHO) classifies meningiomas into grades 1 to 3 based on histologic findings and the presence of brain invasion (2). Earlier studies demonstrated that up to 60% of sporadic meningiomas exhibit biallelic *Neurofibromin 2* (*NF2*) gene inactivation due to chromosome 22 monosomy with concurrent *NF2* point mutations (NF2 meningiomas/tumors) (3, 4). A series of papers subsequently described that non-NF2 meningiomas were subdivided into genomic groups defined by their specific somatic mutations (5, 6). The most frequent coding changes identified in non-NF2 meningiomas were missense mutations in *TNF Receptor Associated Factor 7 (TRAF7), Kruppel-like factor 4 (KLF4)*, and *RAC(Rho family)-alpha serine/threonine-protein kinase 1 (AKT1)* (7, 8). They were respectively found in almost 30%, 12% and 14% of cases (5, 9, 10). Interestingly, mutations in *KLF4* and *AKT1* almost always co-occurred with *TRAF7*, but not with each other (11). Advances in genomic analysis led to additional meningioma classifications based on genome-wide DNA methylation profiling and somatic copy number alterations (12, 13). More recently, whole genome sequencing and transcriptome analysis were combined to propose yet another classification of meningiomas based on molecular profiling into 3 major types (14). Type A tumors carried missense mutations in *TRAF7*, *KLF4*, and *AKT1* without any significant chromosomal alterations, which confirmed previous observations in benign meningiomas (5, 15). Type B meningiomas included non-aggressive tumors primarily distinguished by *NF2* loss (14). Type C meningiomas were more aggressive and displayed a significant burden of chromosomal gains/losses, most commonly loss of both chr22q and chr1p. In contrast to types A and B, which occurred mostly in females, type C meningiomas happened in roughly equal proportion of females and males (14). Additional integration of multiple molecular approaches, including DNA methylation, RNA-seq and cytogenetic profiling, has also been proposed to refine meningioma classification (16, 17). The critical role that molecular profiling may play in meningiomas led to the inclusion of specific high-risk signatures in the 2021 WHO classification (2).

Although molecular profiling of meningiomas is gaining broader traction through identification of its potential clinical implications (12, 17–20), little is known about specific signaling pathways and resulting molecular signatures of different variants of benign meningiomas. Here, we examined transcriptional signatures of four most common benign groups of meningiomas. First, we analyzed meningiomas with *NF2* loss versus tumors with missense mutations in *TRAF7*. Next, we compared NF2 tumors with or without additional chr1p loss. We also examined two groups of TRAF7 tumors carrying additional missense mutations in *AKT1* or *KLF4*. The analysis revealed distinct transcriptional programs specific for each tumor genotype.

## 2 Results

### 2.1 Patient demographics and pathologic characteristics

Due to known gender differences in meningioma occurrence and prognosis, we included only women in this study. Women are diagnosed with meningiomas more frequently and at an older age compared to men (8, 21, 22). Meningiomas in women are more commonly low grade, while those in men are more commonly aggressive. Exclusive selection of meningiomas from women allowed us to remove gender as a potential confounder. Thus, primary samples from meningiomas with WHO grades of 1 (n=18) or 2 (n=2) were selected from twenty female patients (**Table 1**) with a median age of 61 years at the time of surgery (range: 37-77). WHO grades 1 and 2 were included both due to limited numbers of samples available to achieve five samples per group as well as past findings that gene expression is often correlated more closely with genetic profiling than histological grade. Genetic profiling revealed ten tumors with *NF2* loss. Five of these NF2 meningiomas (N2, N4, N5, N9, and N10) had additional cytogenetic changes, including loss of the short arm of chromosome 1 (chr1p), while the other five (N1, N3, N6, N7, and N8) displayed no significant chromosomal instability aside from chr22q monosomy. The ten non-NF2 meningiomas had missense mutations in *TRAF7*. Five of these *TRAF7*-altered meningiomas also contained an E17K mutation in *AKT1* (*AKT1^E17K^*), while the other five *TRAF7*-altered tumors carried additional K409Q mutation in *KLF4* (*KLF4^K409Q^*).

**Table 1:** Clinical features of patients, their meningiomas and identified mutations.

PCA group	Sample ID in PCA Plot	Patient ID	Patient age	CNS WHO grade	Histological Type	Ki-67 labeling index (%)	Mutated genes	Copy number variation	
								Chr 22q loss	Chr 1p loss
NF (*NF2*-altered, *TRAF7-*wildtype)	N1	M-014	51	1	Transitional	3.00	*NF2, CREBZF, JAK2, KDM6B*	Yes	
	N2	M-015	57	1	Meningothelial	2.00	*NF2, COL6A3*	Yes	Yes
	N3	M-018	59	1	Transitional	<1	*NF2, EGFR*	Yes	
	N4	M-024	57	1	Angiomatous	<6	*NF2, NOTCH1, KMT2D, QKI*	Yes	Yes
	N5	M-041	77	1	Meningothelial	5	*NF2, TERT*	Yes	Yes
	N6	M-051	72	1	Meningothelial	3.78	*NF2, ARID2, NOTCH2, PTCH1*	Yes	
	N7	M-062	37	1	Transitional	0.05	*NF2, ATM, WRN*	Yes	
	N8	M-063	73	1	Meningioma	3	*NF2, CDKN2A, ARID1A, SETD2*	Yes	
	N9	M-064	61	1	Fibrous	<1	*NF2, CHEK2, SMARCB1, ARID2, MSH3*	Yes	Yes
	N10	M-071	63	2	Meningothelial	<10	*NF2, CHEK2, NF1, KMT2B, KMT2D, PTCH2*	Yes	Yes
TK (*NF2*-intact, *TRAF7-*mutant*, KLF4-*mutant)	TK1	M-030	51	1	Secretory	1.5	*TRAF7, KLF4, POT1*		
	TK2	M-048	64	1	Secretory	<5	*TRAF7, KLF4, GLI2*		
	TK3	M-066	46	1	Secretory	<2	*TRAF7, KLF4, TCF12*		
	TK4	M-068	61	1	Meningothelial	1.02	*TRAF7, KLF4, POT1, BRAF, GNA11, MLH1*		
	TK5	M-070	64	1	Meningothelial	<1	*TRAF7, KLF4, KMT2D*		
TA (*NF2*-intact, *TRAF7-*mutant*, AKT1-*mutant)	TA1	M-026	58	1	Meningothelial	2.93	*TRAF7, AKT1, TET2*		
	TA2	M-027	55	1	Meningothelial	1.45	*TRAF7, AKT1*,		
	TA3	M-034	54	1	Meningothelial	3.20	*TRAF7, AKT1, FGFR1, KMT2D, TET1*		
	TA4	M-040	65	2	Meningothelial	<1	*TRAF7, AKT1, ARID1A, SETD2*		
	TA5	M-069	53	1	Meningothelial	1.00	*TRAF7, AKT1*		

### 2.2 NF2 and TRAF7 meningiomas display divergent transcriptomes

**Figure 1:**
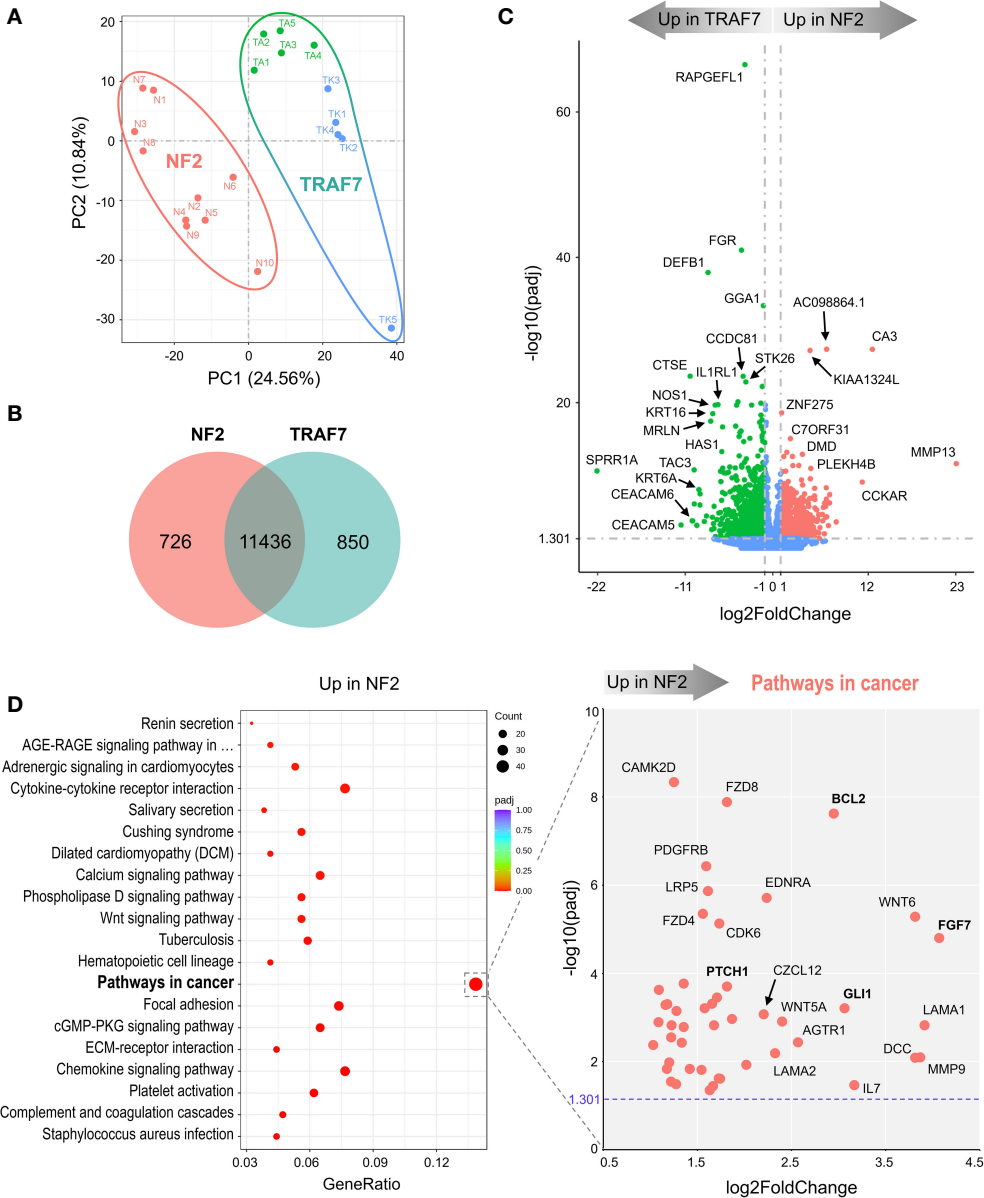
NF2 and TRAF7 meningiomas display divergent transcriptomes. **(A)** PCA plot of RNA-seq analysis in primary meningioma tumors carrying mutations in *NF2* or *TRAF7*. Ten samples were analyzed in each group and are shown in different colors. TRAF7 group of samples combines two subgroups of tumors carrying additional mutations in *KLF4* (blue dots) or *AKT1* (green dots). The sample clusters are circled by lines of corresponding colors. **(B)** Venn diagram showing genes expressed in NF2 and TRAF7 meningiomas. The numbers inside corresponding areas indicate the quantity of non-DEGs or genotype-specific upregulated DEGs identified by RNA-seq. **(C)** Volcano plot visualizing significant DEGs in NF2 versus TRAF7 meningiomas: relative expression (x-axis) vs. statistical significance (y-axis) of difference in mRNA expression. Genes which are not differentially expressed are shown in blue. The directions of increasingly upregulated genes specific for each group are shown by thick grey arrows above the chart with the corresponding tumor group name in it. DEGs with the highest discrepancy in relative expression between two groups are indicated. **(D)** Left panel: Bubble plot of KEGG enrichment analysis of signaling pathways upregulated in NF2 meningiomas versus TRAF7 tumors. Each bubble represents a KEGG pathway. Gene ratio (x-axis) is the propotion of the total genes in a given pathway that are upregulated in the indicated group. Right panel: Scatter plot of genes from the “Pathways in cancer” list. Genes shown in bold are of particular importance.

To understand how tumor-associated mutations cause meningioma growth and to assess the differences between the most common groups of meningiomas, we compared transcriptomes of NF2 and TRAF7 meningiomas (**Supplemental File 1**). The tumors from each group segregated into distinct clusters following principal component analysis (PCA) (**Figure 1A**). We found 1576 differentially expressed genes (DEGs), with 726 upregulated in NF2 meningiomas relative to TRAF7 meningiomas, and 850 upregulated in TRAF7 meningiomas (**Figure 1B**). Although the majority of identified genes were the same between these two groups, the tumors across groups were easily discernable based on gene expression profiles (**Figure S1A**), and expression patterns were highly similar among samples in each group (**Figure S1B**). Both groups expressed high levels of selected meningeal genes (**Figure S2**), with four meningeal genes displaying significantly different expression between groups. Specifically, NF2 meningiomas expressed higher levels of arachnoid *CLDN11* and pial *LAMA2*, while TRAF7 tumors overexpressed dural *MGP* and dural/arachnoid *CRABP2* relative to NF2 tumors.

Further examination revealed the presence of DEGs with large differences in relative expression and high statistical significance between NF2 and TRAF7 tumors (**Figure 1C**). *RAPGEFL1*, encoding RAP guanine nucleotide exchange factor like 1 protein, displayed the highest statistical significance of differential expression in TRAF7 meningiomas compared to NF2 counterparts. Interestingly, TRAF7 meningiomas expressed high levels of biomechanically inducible genes, including keratins *KRT16*, *KRT6A*, and gene encoding IL33 receptor IL1RL1, also known as ST2 (23–25). In addition, neuronal *nitric oxide synthase 1* (*NOS1*) was also highly expressed in TRAF7 meningioma. On the other hand, NF2 meningiomas expressed high levels of genes for muscle specific carbonic anhydrase CA3 and matrix metalloproteinase MMP13 compared to TRAF7 tumors. This analysis highlighted the unique transcriptional profiles of NF2 and TRAF7 meningiomas, indicating that tumor-specific genetic alterations lead to activation of divergent signaling pathways in these tumor cells.

KEGG gene set enrichment analysis found that meningiomas with *NF2* inactivation are enriched for “pathways in cancer”, indicating the susceptibility of NF2 meningiomas to undergo further aggressive evolution (**Figure 1D**, left panel). We plotted the relative expression and adjusted p value of the 47 DEGs upregulated in NF2 meningiomas involved in this pathway (**Figure 1D**, right panel). Interestingly, anti-apoptotic regulator *B-cell CLL/Lymphoma 2* (*BCL2)* and *glioma associated oncogene homolog* 1 *(GLI1)* appeared among a number of other pro-proliferation genes associated with the loss of NF2. Because BCL2 was first identified as a cell death regulator following cloning from B lymphocyte malignancies (26, 27), we examined the expression of lymphocyte markers in both meningioma groups. We found no differentially expressed constitutive B cell (*CD19, CD20, CD22, CD40* and *CD80*) or T cell (*CD3*, *CD4*, and *CD8*) markers between NF2 and TRAF7 tumors (**Supplemental File 1**), suggesting similar levels of lymphocyte infiltration. In contrast, there was a modest but significant increase in relative expression of myeloid markers (*CD14*, *CD33* and *CD74*) in NF2 meningiomas, suggesting higher infiltration of NF2 tumors by myeloid cells. Our analysis of leukocyte activation markers revealed no difference in the relative expression of canonical T lymphocyte activation markers, *IL2RA/CD25, CD40LG/CD154*, and *CD69*, but higher expression of activated antigen presenting cell marker *CD86* in NF2 meningiomas when compared with TRAF7 tumors. Indeed, Gene Ontology (GO) terms analysis revealed that NF2 tumors displayed enrichment of genes in multiple pathways related to leukocyte activation and migration (**Figure S3A**, left panel).

TRAF7 meningiomas were enriched in MAPK (mitogen-activated protein kinases) signaling pathway members (**Figure S3B**, left panel), implying that *TRAF7* mutants may induce expression of MAPK genes to drive meningioma growth. In contrast to NF2 meningiomas, no obvious oncogenes were present among DEGs upregulated in TRAF7 meningiomas within the “MAPK signaling pathway” set, underscoring the non-aggressive nature of TRAF7 meningiomas (**Figure S3B**, right panel). Interestingly, both NF2 and TRAF7 meningiomas possess upregulated genes of canonical members of the fibroblast growth factor (FGF) family. *FGF7* was highly expressed in NF2 meningiomas, while *FGF10, FGF17*, and *FGF1* had increased relative expression in TRAF7 tumors. All of these FGFs bind to FGF receptor 2 (FGFR2), deregulation of which has been observed in many types of cancer (28). GO analyses found enrichment of epidermis development and regulation of cell activation pathways in TRAF7 and NF2 meningiomas, respectively (**Figure S3A**).

### 2.3 Transcriptome analysis of two meningioma subgroups with *NF2* inactivation

**Figure 2:**
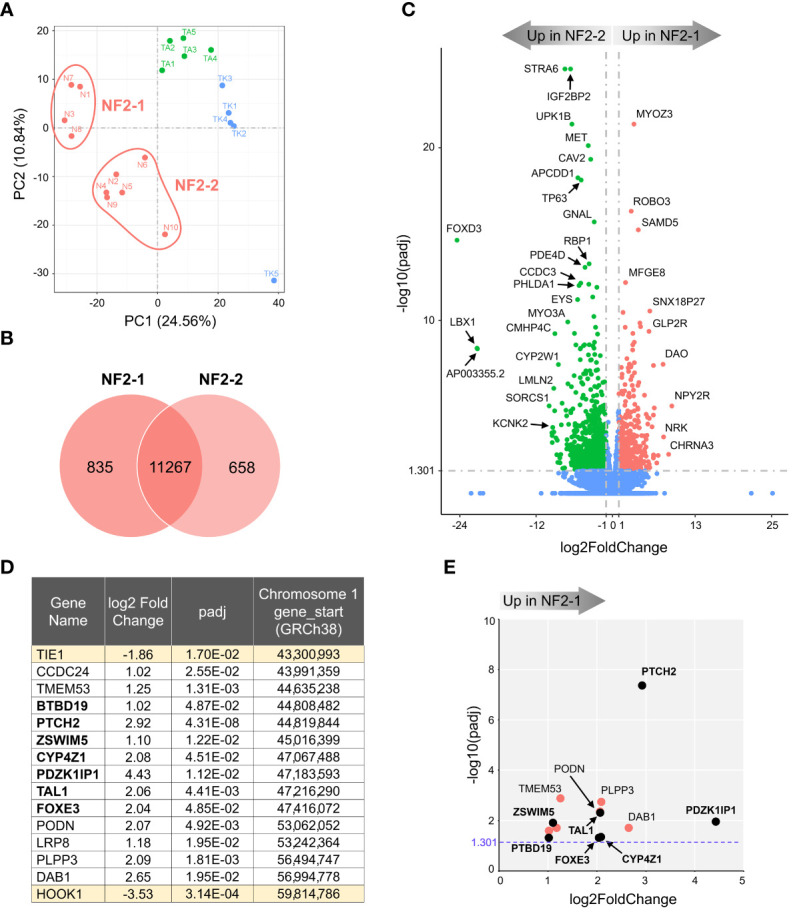
Transcriptome analysis of NF2 meningioma groups. **(A)** Two sub-clusters of NF2 meningioma samples on PCA plot of RNA-seq analysis: four samples in NF2-1 and six samples in NF2-2 groups. **(B)** Venn diagram showing the overlap of DEGs in NF2 meningioma groups. The numbers of shared and tumor group-specific genes are shown inside the corresponding areas. **(C)** Volcano plot visualizing significant DEGs in NF2-1 versus NF2-2 tumors: relative expression (x-axis) vs. statistical significance (y-axis) of difference in mRNA expression. Genes which are not differentially expressed are shown in blue. The directions of increasingly upregulated genes specific for each group are shown by thick grey arrows above the chart with the corresponding tumor group name in it. DEGs with the highest discrepancy between two groups are indicated. **(D)** List of 15 DEGs positioned in missing 16.5 Mb long region from chr1p. Table shows the relative expression (log2FoldChange) of a given gene in NF2-1 versus NF2-2 tumors, statistical significance (padj), and gene start location on chr1p (from Reference GRCh38.p14 Primary Assembly). DEGs located in the smallest region of overlapping chr1p deletions are shown in bold font. **(E)** Scatter plot of DEGs as listed in table in **(D)**: relative expression (x-axis) vs. statistical significance (y-axis) of difference in mRNA expression. DEGs located within the smallest 2.8 Mb region of overlapping chr1p deletions are shown as black dots and marked in bold font.

We next compared mRNA expression profiles of NF2 meningioma subgroups. The PCA plot in **Figure 2A** suggests that NF2 tumors comprise two distinct subgroups, NF2-1 and NF2-2. Interestingly, this segregation coincides with genetic characteristics of tumor samples based on the loss of chr1p. NF2-1 group included four meningiomas with intact chromosome 1, while NF2-2 group included five tumors with chr1p loss and one (N6) meningioma with intact chromosome 1. There were 835 and 658 DEGs specifically upregulated in NF2-1 and NF2-2 groups relative to each other (**Figure 2B**). A tumor suppressor gene *FOXD3* (29) had the highest significant change in relative expression in NF2-2 meningiomas compared to NF2-1 tumors (**Figure 2C**). Curiously, *FOXD3* is located on chr1p, part of which is missing in this group of tumors. However, its position (1p32.1-1p31.2) is close to but outside of a previously determined smallest region of overlapping (SRO) chr1p deletions in meningiomas on 1p33-1p34 (30). These results suggested that genomic changes introduced by SRO deletion may contribute to aberrant expression of SRO proximal genes. It is also interesting that the relative expression level of antisense long non-coding RNA (lncRNA) *FOXD3-AS1* was also significantly increased in NF2-2 meningiomas compared to NF2-1 tumors (**Supplemental File 2**). Considering recent findings that *FOXD3-AS1* is required for cell pluripotency and cancer development (31), our observations of high concurrent *FOXD3* and *FOXD3-AS1* relative expression in NF2-2 meningiomas merits further investigation.

KEGG gene enrichment analyses revealed that both NF2 subgroups had enrichment of a “neuroactive ligand-receptor interaction pathway” (**Figure S4A**). NF2-1 tumors additionally showed enrichment of the pathways involved in “transcriptional misregulation in cancer” and “regulation of actin cytoskeleton”. In contrast, the “PI3K/AKT signaling pathway” had the second highest proportion of genes upregulated in NF2-2 tumors. GO database analyses ranked the “extracellular matrix” pathway as the most enriched in NF2-1 group, while the “angiogenesis” set dominated in NF2-2 tumors (**Figure S4B**).

The SRO of chr1p deletions was determined to be an approximately 2.8 megabases (Mb) long fragment and includes genes from *PLK3 (CNK) to TRABG2b (RH68723)* (30). We examined DEGs in this region and surrounding areas and selected a chromosome fragment enclosed by the two nearest genes upregulated in NF2-2, from *TIE1* to *HOOK1*. This fragment was 16.5 Mb long and contained 13 DEGs upregulated in NF2-1 compared to NF2-2 (**Figure 2D**). Seven of those DEGs were located inside the 2.8 Mb SRO region (30). For better visual display of expression profiles of DEGs located within the 16.5 Mb fragment, their relative expression versus statistical significance was plotted in **Figure 2E**. Patched 2 receptor gene (*PTCH*2), a tumor suppressor in the Sonic Hedgehog (SHH) signaling pathway, appeared to be the most significantly upregulated gene in this group, implying that decreased expression of *PTCH2* due to chr1p loss may result in increased aggressiveness of NF2-2 meningiomas.

### 2.4 Divergent transcriptomes of KLF4 and AKT1 meningiomas

**Figure 3:**
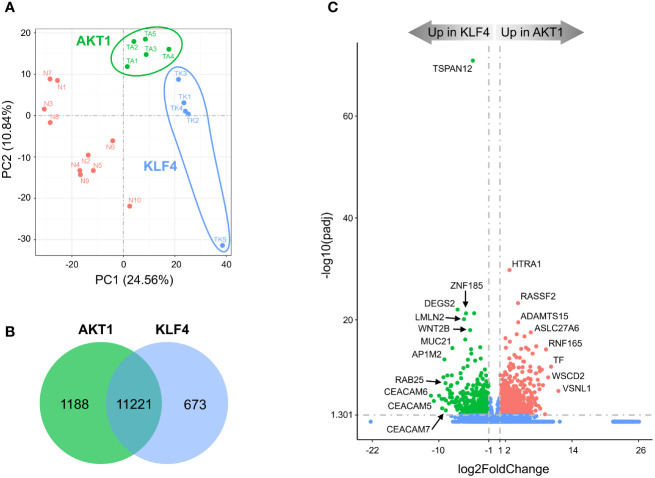
Divergent transcriptomes within TRAF7 group of tumors: AKT1 and KLF4 meningiomas. **(A)** PCA plot of RNA-seq analysis in primary TRAF7 meningiomas carrying additional mutations in AKT1 or KLF4. Five samples were analyzed in each group. Sample clusters are shown by different colors (KLF4 in blue, AKT1 in green) and circled by corresponding color lines. **(B)** Venn diagram showing the overlap of DEGs in AKT1 and KLF4 meningiomas by RNA-seq analysis. Common and tumor genotype-specific genes are depicted by numbers inside the corresponding circles. **(C)** Volcano plot visualizing significant DEGs in AKT1 vs. KLF4 meningiomas: relative expression (x-axis) vs. statistical significance (y-axis) of difference in mRNA expression. Genes which are not differentially expressed are shown in blue.

In agreement with observed mutations in *AKT1* and *KLF4*, TRAF7 meningiomas segregated into two distinct clusters (**Figure 3A**). AKT1 meningiomas displayed increased expression of 1188 genes and decreased expression of 673 genes relative to KLF4 tumors (**Figure 3B**). The most significant gene overexpressed in KLF4 meningiomas relative to AKT1 meningiomas was tetraspanin *TSPAN12* (**Figure 3C**), which encodes a member of the transmembrane 4 superfamily. Interestingly, DEGs with the highest relative expression in KLF4 tumors were carcinoembryonic antigen-related cell adhesion molecule 6 (*CEACAM6*) and *CEACAM5*, also known as CD66c and CD66e. These surface glycoproteins are normally expressed in gastrointestinal tissue during embryonic development, but their production stops before birth (32). Importantly, they are highly expressed in human carcinomas, including colon, ovarian, pancreatic, non-small cell lung, head and neck, cervical, uterine and breast cancers (33).

KEGG analysis revealed that, similar to NF2 groups, AKT1 tumors were enriched for the “neuroactive ligand-receptor interaction” pathway (**Figure S5A**). In comparison, the most significantly enriched signaling pathway in KLF4 tumors was “RAP1 signaling pathway”. RAP1 is a small GTPase-activating protein involved in regulation of vascular permeability (34). Our results also revealed that the vascular endothelial growth factor A (*VEGFA*) was a part of the “RAP1 signaling pathway” profile (**Figure S5A**, right panel), and its high expression is known to be associated with peritumoral brain edema (35). We found that *VEGFA* mRNA expression was almost 3-fold higher in KLF4 meningiomas compared to AKT1 tumors (**Supplemental File 3**), in agreement with previous studies (36, 37). Moreover, TRAF7 meningiomas express >5-fold higher levels of *VEGFA* compared to NF2 tumors (**Figure S3B**, right panel and **Supplemental file 1**), suggesting that *VEGFA* together with other DEGs from the RAP1 signaling pathway may be responsible for the secretory phenotype of KLF4 meningiomas. In addition, both AKT1 and KLF4 meningiomas demonstrated trending enrichment of the “PI3K/AKT signaling pathway”. These results are expected for AKT1 tumors, which harbor activating E17K mutation (AKT1^E17K^), and suggest that changes induced by KLF4^K409Q^ at least partially resemble AKT1 activation. These results also suggest that AKT1 and KLF4 tumors are somewhat similar to NF2-2 meningiomas, where an activation of the PI3K/AKT signaling pathway was also observed (**Figure S4A**). GO analysis uncovered that the “extracellular matrix” gene set was the most representing in upregulated DEGs in AKT1 meningiomas, while KLF4 tumors upregulated DEGs important for “epidermis development” and “cell motility” (**Figure S5B**). Other GO sets enriched in KLF4 tumors included, regulation of locomotion and cell motility, in agreement with the expression of genes induced by RAP1 signaling.

### 2.5 TRAF7 deficiency upregulates expression of *KRT6A/16*, *IL1RL1*, and *AQP3* genes

**Figure 4:**
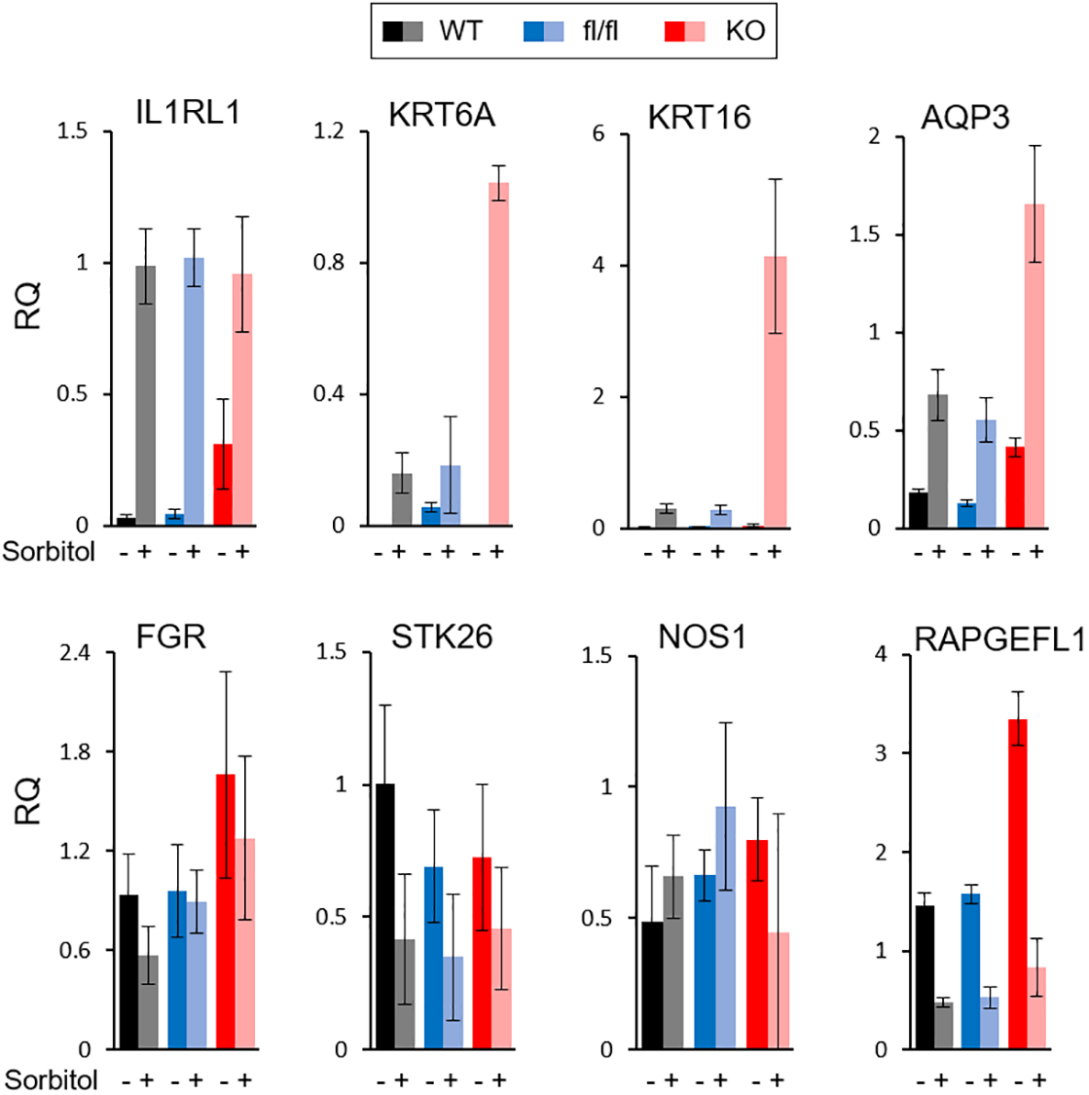
Analysis of gene expression in TRAF7 deficient cells. mRNA expression analysis of several TRAF7-dependent DEGs (as labeled above each graph panel) in TRAF7^+/+^ (WT), TRAF7^fl/fl^ (fl/fl), and TRAF7^-/-^ (KO) mouse embryonic fibroblasts un-stimulated (-) or stimulated (+) with 0.2M sorbitol as marked. Total RNA was purified 16 hours post-stimulation and mRNA was amplified by RT-qPCR using GAPDH as internal control. Bars represent mRNA fold change over mRNA value in un-stimulated WT. Data are presented as relative quantity (RQ) Mean ± SE.

All of the described TRAF7 mutations in meningioma are missense, with no nonsense or frameshift mutations. Although they are recurrent, they are not limited to a single amino acid position, as happens with AKT1 or KLF4 mutations, but distributed across a sizeable C-terminal part of the protein. These results suggest that TRAF7 mutations most likely do not cause gain-of-function, as AKT1^E17K^ and KLF4^K409Q^, but instead are loss-of-function and/or dominant negative. Since all other TRAF proteins are known to form homo- or hetero-trimers (38), the mutant TRAF7 protein may also trimerize with the wild type (WT) protein and result in at least partially inactive complexes with dominant-negative function as was shown for other multimeric proteins with various missense mutations (39). To investigate whether high expression of genes in TRAF7 meningiomas resulted from loss of TRAF7 function, we examined expression of several of them in TRAF7-deficient mouse embryonic fibroblasts (MEFs). We chose upregulated genes with the high statistical significance and relative expression (**Figure 1C**) and tested their expression in MEFs following hyper-osmotic stress induced by high concentration of sorbitol. The expression of *IL1RL1* was significantly higher in untreated *TRAF7*-deficient (*TRAF7^-/-^*) MEFs compared to untreated WT or *TRAF7^fl/fl^* cells (**Figure 4**). However, sorbitol treatment induced similar levels of *IL1RL1* expression in cells of all three genotypes. These results indicate that TRAF7 inhibits *IL1RL1* expression in untreated cells and also suggest that the lack of TRAF7 imitates hypertonic stress conditions, resulting in higher expression of *IL1RL1*, a biomechanically induced gene (23). Next, we examined the expression of several selected TRAF7 meningioma-specific DEGs in *TRAF7^-/-^* MEFs. Although we found no difference in the expression of *FGR*, *STK26*, *CTSE*, and *NOS1* between WT, *TRAF7^fl/fl^* and *TRAF7^-/-^* MEFs (**Figure 4**), the expression of *AQP3* in *TRAF7^-/-^* cells was increased in untreated *TRAF7^-/-^* MEFs. Moreover, *KRT6A*, *KRT16*, and *AQP3* were highly induced in sorbitol-treated *TRAF7^-/-^* MEFs compared to WT or *TRAF7^fl/fl^* cells (**Figure 4**). Interestingly, *KRT6A*, *KRT16*, and *AQP3* were also termed biomechanically responsive genes because all of them are induced in response to acute skin injury (24, 25, 40).

## 3 Discussion

### 3.1 Transcriptional signatures of NF2 meningioma groups

The *NF2* gene, on the long arm of chromosome 22, encodes a 69 kDa protein called neurofibromin 2 (also called merlin or schwannomin) (41). Neurofibromin 2 is an intracellular scaffold protein that links actin filaments, transmembrane receptors and intracellular proteins. It is a tumor suppressor and its biallelic inactivation results in several types of central and peripheral nervous system tumors, including schwannomas, ependymomas, meningiomas, and others (42). Consistently, conditional knockout mice with Cre-mediated excision of *NF2* in schwann cells developed schwannomas, schwann cell hyperplasia, cataract, and osseous metaplasia (43). Despite the well-known *NF2* role as a tumor suppressor, no new chemotherapeutic approaches have yet been developed, probably due to its broad involvement in different signaling pathways and interaction with multiple protein partners (44).

Here, we provide evidence that *NF2*-deficient meningiomas express significantly higher levels of widely recognized oncogenes *BCL2* and *GLI1* compared to meningiomas with missense *TRAF7* mutations (**Figure 1D**, right panel). Although *BCL2* was originally identified as an anti-apoptotic gene in B-cell lymphomas (45), it was later shown to suppress apoptosis in a variety of cell systems, including neural and other cell types (46). On the other hand, aberrant activation of the SHH/PTCH1 signal transduction pathway in cancer cells triggers nuclear translocation of GLI transcription factors and overexpression of *BCL2* (47–49). Consistent with our results, it was shown that a WHO grade 1 meningioma cell line with *NF2* loss, Ben-Men-1, expressed much higher levels of *BCL2* mRNA compared to primary meningeal cells (50), making infiltrating activated leukocytes an unlikely contributor to high expression of *BCL2* and *GLI1* in NF2 meningiomas.

Our comparison of two subgroups of NF2 meningiomas revealed that NF2-2 tumors with chr1p loss have a much lower expression of tumor suppressor *PTCH2* compared to NF2-1 tumors with intact chr1p (**Figure 2E**), suggesting that a decreased expression of this gene may underlie a more aggressive nature of aggressive meningiomas with significant chromosomal losses, including chr22q and chr1p. Previously, it was shown that an inactivating missense mutation in a single allele of *PTCH2* caused a pleiotropic, autosomal dominant basal cell syndrome (51, 52). However, patients with mutated *PTCH2* displayed milder phenotypes of Gorlin syndrome when compared against *PTCH1* and *SUFU*-related diseases (53). In our cohort, N6 meningioma with intact chr1p (**Table 1**), but where one copy of *PTCH1* has a 104G>A mutation resulting in R35Q amino acid change in PTCH1, clustered on a PCA plot with tumors which lost chr1p (**Figure 2A**). It is possible that inactivation of a *PTCH1* copy has a similar functional effect as the loss of chr1p given that the combination of mutations in both *PTCH1* and *PTCH2* promoted a dramatic increase in the incidence of tumorigenesis (54). Taken together, these results suggest that dysregulation of the Hedgehog signaling pathway and subsequent increased expression of *GLI1* and *BCL2* may play a role in the development of meningioma as is the case in other cancers (55). These results suggest that therapeutic targeting of the GLI1-BCL2 pathway may be a rational exploratory step in the development of efficacious chemotherapeutic approaches for the treatment of merlin-deficient tumors.

Our results demonstrated that the loss of chr1p also significantly increased the expression of *FOXD3* and *FOXD3-AS1* in NF2-2 meningiomas compared to NF2-1 tumors. FOXD3 was discovered as a pioneer transcription factor with a unique ability to bind to condensed chromatin and initiate transcriptional activation of target genes (56–58). Later, it was shown to be required for maintaining pluripotency in mouse embryonic stem cells (59). *FOXD3* as well as *FOXD3-AS1* play roles in the initiation of progression of several diseases (60, 61). Considering that *FOXD3* is located just outside of the 2.8Mb long NF2-2 meningioma SRO, these observations indicate that the deletion of chr1p SRO may contribute to deregulation of proximal genes. This also suggests a need for further detailed investigation of chr1p deletions and their role in gene expression.

Prior work has shown that primary atypical meningiomas, comprised mostly of *NF2* mutants with genomic instability or recurrent *SMARCB1* mutations, display a hypermethylated phenotype due to increased polycomb repressive complex 2 (PRC2) activity (13). Benign type B meningiomas with *NF2* loss but no other chromosomal abnormalities have less PRC2 complex repressor activity compared to more aggressive type C meningiomas that lack both chr22q and chr1q (14). Similarly, proliferative meningiomas with *NF2* loss but no chromosomal instability demonstrate lower methylated status when compared to other molecular groups (17). Since methylation profiling of meningiomas in our study was not assessed, we examined the relative expression of nine genes shown to be the PRC2 complex targets that were upregulated in type B meningiomas (14). Our results revealed that five of these nine genes, *RBP4*, *ELN*, *HOXB2*, *ATOH8*, and *SFRP4*, were upregulated in the NF2-1 group compared to the NF2-2 group (**Supplemental File 2**).

Type C meningiomas, characterized by *NF2* loss and genomic instability, were proposed to be deficient in the repressive dimerization partner, RB-like, E2F and multi-vulval class B (DREAM) complex, a master regulator of gene expression (14). The authors found increased expression of the DREAM complex partners *FOXM1* and *MYBL2* in those tumors compared to type B meningiomas, resulting in activation of the DREAM complex. In our study, the NF2-2 subgroup displayed a 4-fold higher expression of *MYBL2* compared to NF2-1 subgroup, but there was no difference between the NF2 subgroups in the expression of *FOXM1* (**Supplemental File 2**). Furthermore, of the four DREAM complex target genes shown to be upregulated in type C tumors (14), only *PBK* was higher in our NF2-2 meningiomas, while the expression of the 3 other genes (*TTK*, *MELK*, and *CDK1*) was not significantly different between the subgroups (**Supplemental File 2**). Taken together, these results indicate that no solid conclusions about the repressive DREAM complex function in NF2-2 meningiomas with the loss of chr22q and chr1p could be drawn, potentially due to a lack of power (14).

### 3.2 Transcriptional signatures of TRAF7 meningioma groups

TRAF7 is a unique member of TRAF family (62). It lacks the TRAF domain, and instead contains a WD40 domain (63). Like other TRAF proteins, TRAF7 may form a trimer through a coiled-coil (CC) region (38). Through the WD40 domain, TRAF7 specifically interacts with a number of proteins including mitogen-activated protein kinase kinase kinase 3 (MAP3K3/MEKK3) (64, 65), transcriptional activator MYB (c-Myb) (66), dual specificity mitogen-activated protein kinase kinase 5 (MAP2K5/MEK5) (67), Roundabout homolog 4 (ROBO4) (68), and NF-κB essential modulator (NEMO) (69). Here, TRAF7 meningiomas expressed a high number of specific DEGs, including *RAPGEFL1*, *KRT16*, *KRT6A*, *IL1RL1*, *NOS1*, and others (**Figure 1B**). Gene enrichment analysis revealed DEGs involved in “MAPK signaling pathway” and “tight junction”, but no clear subgroup-specific dominant equivalent pathway as “pathways in cancer” in NF2 tumors (**Figure S3B**). One of the reasons might be that *TRAF7* mutants expressed in hemizygous meningioma cells foster partial loss of normal TRAF7 function through the formation of dysfunctional hetero-trimers consisting of normal and mutant TRAF7 proteins. In agreement, *TRAF7*-deficient MEFs also had an increased expression of several investigated TRAF7 meningioma signature genes, including *IL1RL1*, *KRT16, KRT6A,* and *AQP3* (**Figure 4**). Neomorphic function of mutant TRAF7 homo- or heterotrimers is also a possibility, but it seems unlikely that identified missense *TRAF7* mutations, which occur at different amino acid positions throughout CC and WD40 domains, would generate the same tumor growth signal.

Interestingly, the mutated allele KLF4^K409Q^ always occurs together with *TRAF7* missense mutations and is the same in all affected patients (5), suggesting a potential neomorphic “gain-of-function” role of the mutant protein. Indeed, KLF4 meningiomas share a unique secretory phenotype, characterized by glandular lumina with secretory globules, and tend to cause disproportional peritumoral edema (70, 71). Moreover, we recently demonstrated the molecular mechanism of how KLF4^K409Q^ drives meningioma development (72). The K409K mutation in the DNA-binding domain of KLF4 alters its DNA recognition preference, causing it to bind to a novel consensus sequence and drive transcription of new set of genes. In contrast to KLF4^K409Q^, which was only found in grade 1 meningiomas and in low-grade intraductal papillary mucinous neoplasms (IPMNs) (73), AKT1^E17K^ occurred in many other types of cancer, including breast, lung, ovarian, colorectal and pancreatic carcinomas as well as melanomas and glioblastomas (74). Like K409Q in KLF4, E17K in AKT1 is the same in all tumors and has a clearly defined molecular mechanism of action. AKT1^E17K^ leads to increased binding of phosphatidylinositol-3,4,5-trisphosphate (PIP3) ligand and increased localization to the plasma membrane, where it stimulates downstream subsequent PI3K pathway (75). Consistently, AKT1^E17K^ was associated with reduced time to meningioma recurrence and PI3K/AKT/mTOR oncogenic pathway, which is the most frequently mutated pathway in human cancer (76). Since the majority of TRAF7 tumors harbor a gain-of-function mutation in a single amino acid position in either AKT1 or KLF4, AKT1^E17K^ and KLF4^K409Q^ appear to be the driving force behind TRAF7 meningioma growth. On the other hand, there are no reports of meningiomas with only *AKT1* or *KLF4* mutations, suggesting that a missense *TRAF7* alteration is pre-requisite for *AKT1* or *KLF4* mutations to cause tumor. Thus, TRAF7 controls one of the cellular homeostasis checkpoints and its mutation makes meningeal cells susceptible to proliferation caused by an additional “partner-in-crime” mutation such as AKT1^E17K^ or KLF4^K409Q^.

### 3.3 Conclusion

**Figure 5:**
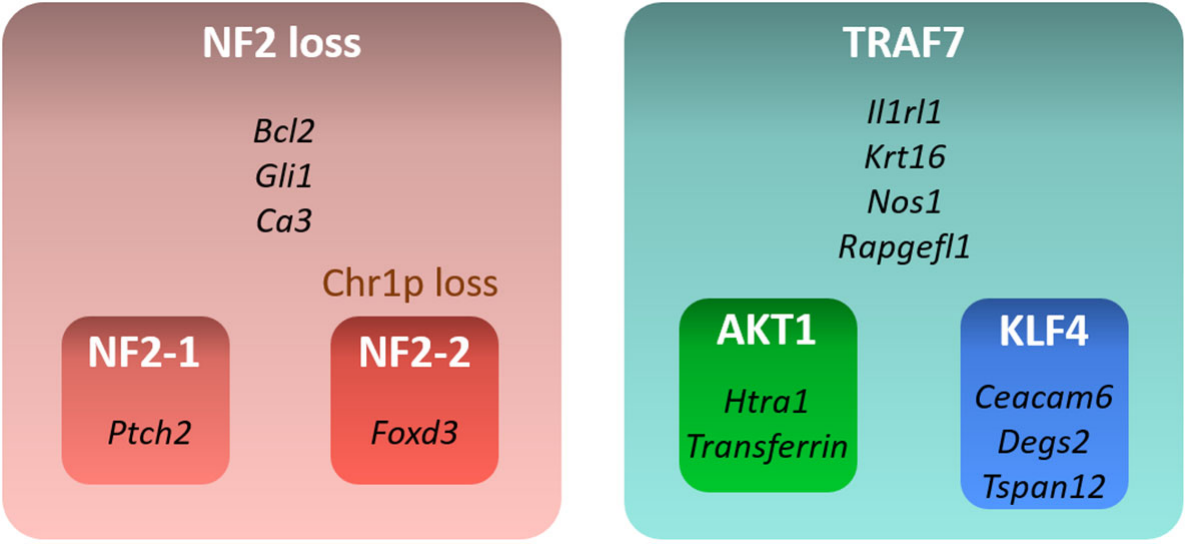
Summary of gene signatures for different benign meningioma subtypes. Only highly expressed and significant genes specific for each subtype are shown.

In this study, we examined transcriptional profiles of four groups of benign meningiomas harboring the most frequent DNA alterations. Our analysis revealed specific gene expression profiles of each of these groups (**Figure 5**). NF2 meningiomas expressed high levels of *BCL2*, *GLI1*, and *CA3*, while TRAF7 tumors had high expression of *IL1RL1*, *KRT16*, *NOS1*, and *RAPGEFL1*. The expression of *FOXD3* and *PTCH2* were upregulated in NF2 meningiomas with or without chr1p loss, respectively. AKT1 meningiomas displayed high relative levels of *HTRA1* and transferrin genes relative to KLF4 tumors, while KLF4 tumors overexpressed *CEACAM6*, *DEGS2*, and *TSPAN12* relative to AKT1 tumors.

### 3.4 Limitations of study

Here, we identified signature genes in meningioma groups with the most commonly observed mutational profiles by using transcriptome analyses in tumors from a small cohort of patients. Our study only unveils differentially expressed genes and lists associated signaling pathways, which may potentially contribute to meningioma growth. Gene expression profiles resulting from genetic mutations do not imply direct associations with previously described epigenetic modifications of meningioma groups. The analysis of cellular meningioma diversity and the mechanistic role of signature genes in tumor development is a subject for future studies and outside the scope of the present study.

## 4 Materials and methods

### 4.1 Patients and sample collection

All procedures were approved by the institutional review board (IRB) of the University of Oklahoma Health Sciences Center (OUHSC). Twenty tumor samples from 20 patients (one sample/patient) diagnosed with meningioma at University of Oklahoma Medical Center were included in the study. Clinical information, including demographics, data on age, and tumor location, was collected by retrospective chart review accessed from the historical archive of the hospital.

### 4.2 Histopathologic grading and genetic profiling

Following routine pathology processing, resected meningiomas were assigned a histopathologic grade according to the revised 5^th^ edition of the WHO Classification of Tumors’ of the CNS (2). Immunohistochemical staining for glial fibrillary acid protein and epithelial membrane antigen were performed on a case-by-case basis as deemed necessary for diagnostic evaluation. Ki-67 immunostaining was performed on at least one block in all cases. All samples were analyzed, graded, and independently confirmed by two staff neuropathologists.

For genetic profiling of tumor, specimens were sent to the Mayo Clinic Laboratories. Somatic mutations and gene rearrangements were examined by the NONCP panel, while copy number imbalances and loss of heterozygosity were estimated by CMAPT panel. For RNA extraction, resected tissues were immediately submerged in RNAlater^®^ Solution, kept at room temperature for 24 hours, and stored frozen long term (Fisher Scientific, AM7023).

### 4.3 RNA-seq and differential expression analysis

Total RNA was extracted from tumors saved in RNAlater^®^ Solution (Fisher Scientific, AM7023) with the RNeasy Plus mini kit (QIAGEN, 74136) with QIAshredder (QIAGEN, 79656). Preparation of cDNA libraries and sequencing was conducted by Novogene Co., LTD (Beijing, China). Significant DEGs were defined as those that had both an absolute log2FoldChange ≥ 1 as well as a false discovery rate adjusted p-value ≤ 0.05 for each comparison independently.

### 4.4 Mice and preparation of mouse embryonic fibroblasts

All housing and experimental use of mice were carried out in AAALAC-accredited facility in accordance with United States federal, state, local, and institutional regulations and guidelines governing the use of animals and were approved by OUHSC Institutional Animal Care and Use Committee. MEFs were prepared from WT and TRAF7^fl/fl^ embryos as described in (77) and TRAF7 gene was excised with Ad(RGD)-mCherry-iCre adenovirus (Vector lab; #1771) according to the manufacturer instructions. WT, TRAF7^fl/fl^, and TRAF7^-/-^ cells were starved for 4 hours before being treated with 0.2M sorbitol overnight (78). Total RNA was extracted with the RNeasy Plus mini kit (QIAGEN, 74136) with QIAshredder (QIAGEN, 79656) according to the manufacturer’s instructions.

### 4.5 Quantitative PCR

Total cell RNA was used to measure gene mRNA levels by real-time qPCR. Reverse transcription and cDNA amplification were performed in one tube using qScript™ XLT One-Step RT-qPCR ToughMix^®^, Low ROX™ (VWR Quanta Biosciences™, 95134) on an Applied Biosystems 7500 Fast Real-Time PCR System (Fisher Scientific). Sample reactions were run in 3-6 replicates. Each mRNA analysis was run in a DuPlex PCR reaction with GAPDH as an internal control. Standard curves for each gene were run to verify the linear range of amplification. Input RNA was kept under 200 ng per reaction to stay within the linear range for GAPDH levels.

All data were analyzed in Microsoft Excel with the built-in analysis methods. TaqMan assays used for RT-qPCR are as follows (m – mouse assays):

mGAPDH-Fwd CCTGTTGCTGTAGCCGTATT

mGAPDH-Rev AACAGCAACTCCCACTCTTC

mGAPDH Probe TTGTCATTGAGAGCAATGCCAGCC

mIL1RL1-Fwd GCGGAGAATGGAACCAACTA

mIL1RL1-Rev TGTGTGGTTGTATGGAGGATTT

mIL1RL1 Probe ACGGCCACCAGATCATTCACAGTT

mSTK26-Fwd CCACCATGCTCAAGGAGATT

mSTK26-Rev CACCTTGTTCTGAAAGCAAGAC

mSTK26 Probe TCCACCGAGACATTAAAGCTGCCA

mNOS1-Fwd GAGAAATTCGGCTGTGCTTTG

mNOS1-Rev GACTTGCGGGAGTCAGAATAG

mNOS1 Probe ACAAGGTCCGATTCAACAGCGTCT

mKRT16-Fwd TGAGATGAGGGACCAGTATGA

mKRT16-Rev TGCGGTTGCTCTGGATTAG

mKRT16 Probe ACATCTCTGCGGTTCTTCTCTGCC

mFGR-Fwd GTGTCGGAGGAACCCATTTAT

mFGR-Rev GTTCTGACCTTCTCGATCCTTTAG

mFGR Probe TCATGTGCTATGGTAGCTTGCTGGA

mRAPGEFL1-Fwd CCCTCATCCTTGTAGCTGTT

mRAPGEFL1-Rev GCAAATAGGTGGCTGTTGATAC

mRAPGEFL1 Probe TTCCTCTGGAGAGAAGGTCCTCCT

mAQP3-Fwd TGGAATCTTTGCCACCTATCC

mAQP3-Rev TGGCCAGTACACACACAATAA

mAQP3 Probe TGATCAGTTCATAGGCACAGCCGC

mKRT6A-Fwd GGAAATTGCCACCTACAGGA

mKRT6A-Rev GACTGCACCACAGAGATGTT

mKRT6A Probe ACCATTCAACCTGCACTCCTCTCC

## Data availability statement

Most of the data generated or analyzed during this study are included in this published article and its supporting information file. The unprocessed RNA-seq raw and processed data files have been deposited on NCBI Gene Expression Omnibus (https://www.ncbi.nlm.nih.gov/geo/query/acc.cgi?acc=GSE221429) and are freely available. Further information and requests for materials should be directed to and will be fulfilled by the lead contact, ID (ian-dunn@ouhsc.edu).

## Ethics statement

All procedures were approved by the institutional review board (IRB) of the University of Oklahoma Health Sciences Center (OUHSC). The patients/participants provided their written informed consent to participate in this study.

## Author contributions

ET, AT, and ID designed the study, performed experiments. LG analyzed the data and provided expertise. ET drafted the manuscript. TS and SH collected clinical data. ST, AT, LG, WB, and ID proofread, finalized and approved of the final manuscript. All authors contributed to the article and approved the submitted version.
