# Supplementary material for: Specific gene expression signatures of low grade meningiomas
Source: Front Oncol. 2023 Mar 1;13:1126550. doi: 10.3389/fonc.2023.1126550 (PMC10016690; doi:10.3389/fonc.2023.1126550)
Supplement: Supplementary file 1 [file Presentation_1.pdf]

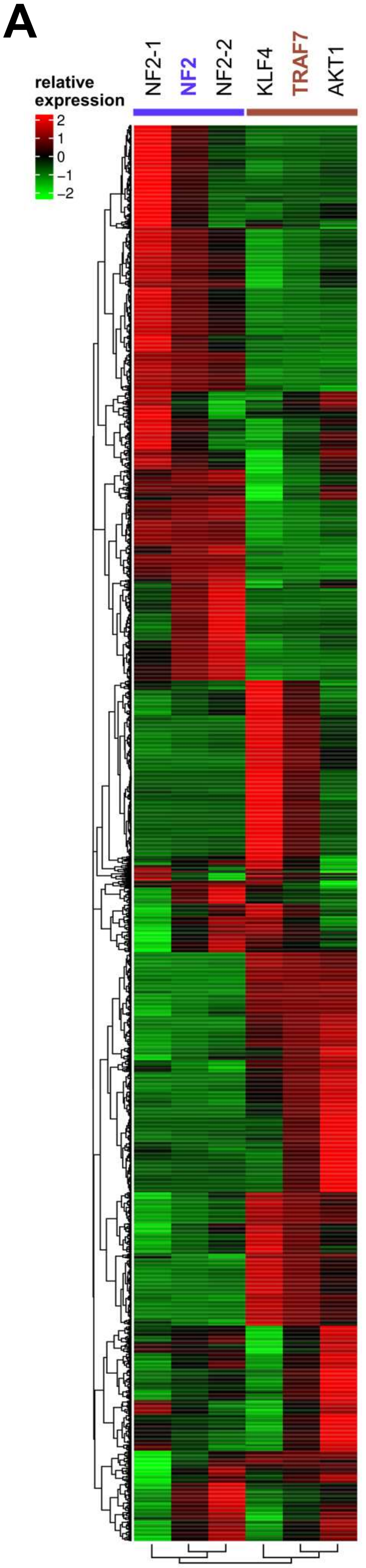

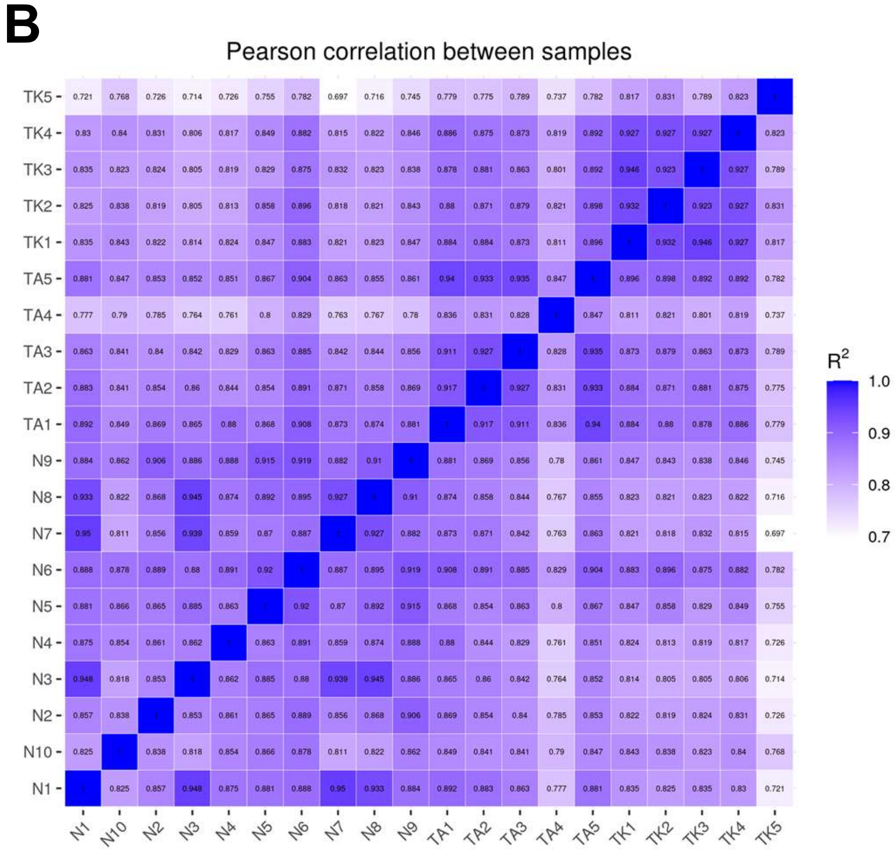

Supplementary Figure 1.
A. Hierarchical clustering of DEGs in groups between NF2 and TRAF7 meningioma samples. Columns corresponding to each meningioma group are indicated at the top. Green and red colors indicate low and high relative mRNA expression levels, respectively. **B.** Heat map of Pearson correlation coefficient between all tested meningioma samples. Each grid in the figure represents the correlation between two samples; different colors represent the relative size of correlation coefficients between samples.

A

Gene Name	NF2 FPKM	TRAF7 FPKM	log2 Fold Change	pvalue	padj	Meningeal layers	Reference
**MGP**	**10,752.87**	**29,889.29**	**-1.47478**	**1.02E-07**	**5.36E-06**	Dura	https://pubmed.ncbi.nlm.nih.gov/32634398/
**CRABP2**	**4,446.73**	**10,113.13**	**-1.18543**	**0.002126**	**0.017225**	Arachnoid/Dura	
CDH1	7,242.84	5,702.79	0.344577	0.269899	0.492371	Arachnoid	
**CLDN11**	**7,288.29**	**595.86**	**3.611915**	**1.26E-09**	**1.20E-07**		
ALDH1A2	1,132.91	2,382.76	-1.07247	0.279203	0.502553		
**LAMA2**	**287.29**	**57.55**	**2.324769**	**0.000579**	**0.006479**	Pial	
S100A6	2,964.48	3,735.93	-0.33311	0.252147	0.474425		
COL1A1	78,490.41	36,900.89	1.08884	0.01669	0.077689		
NGFR	392.53	407.23	-0.05151	0.900515	0.95432		
DCN	23,003.39	20,061.54	0.197392	0.775083	0.887289	Mesenchymal	https://pubmed.ncbi.nlm.nih.gov/35534852/
PECAM1	1,979.87	1,481.62	0.420478	0.180215	0.385769	Endothelial	
CDH5	2,466.85	1,948.98	0.339674	0.376956	0.600569		

B

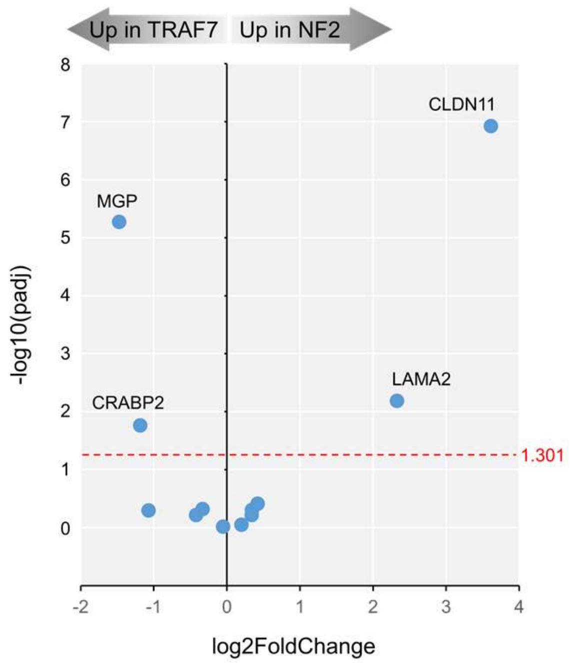

Supplementary Figure 2.
A. List of 12 genes expressed in different meningeal cells. Table shows Fragments Per Kilobase Million (FPKM), relative expression (log2FoldChange), significance (p value) and p adjusted (padj) of expression in NF2 versus TRAF7 tumors. Statistically significant DEGs are shown in bold. **B.** Scatter plot of DEGs as listed in table in (A): magnitude of difference in expression (x-axis) vs. statistical significance (y-axis). Only statistically significant DEGs are labeled.

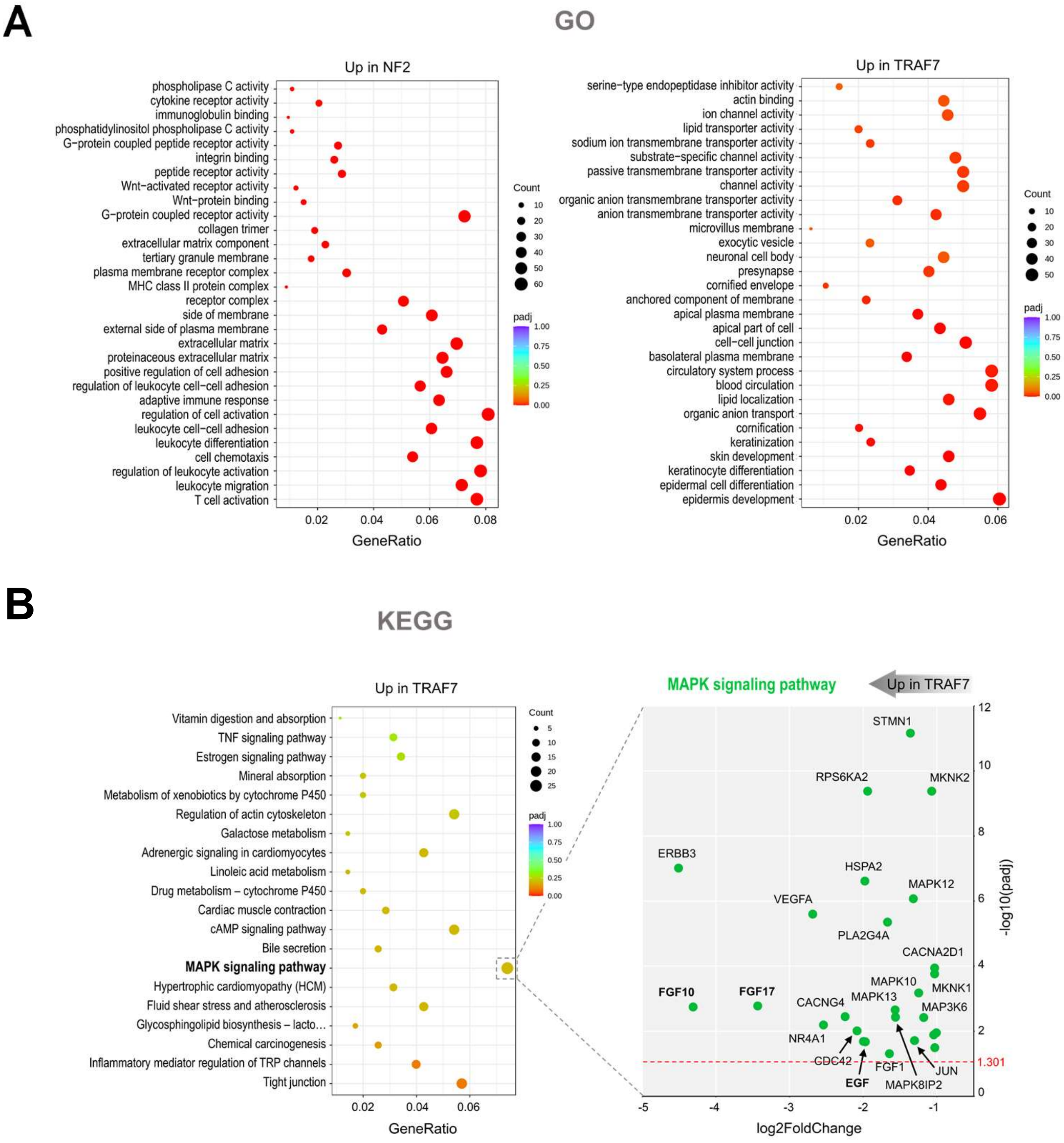

Supplementary Figure 3.
A. Bubble plot of GO pathway enrichment analysis results for DEGs upregulated in NF2 (left panel) and TRAF7 (right panel) meningiomas. Gene ratio (x-axis) is the percentage of significant genes over the total genes in a given pathway. **B.** Left panel: Bubble plot of KEGG enrichment analysis of signaling pathways upregulated in TRAF7 meningiomas. Each bubble represents a KEGG pathway. Right panel: Scatter plot of genes involved in top “MAPK signaling pathway” shown as relative expression (x-axis) vs. statistical significance (y-axis).

A

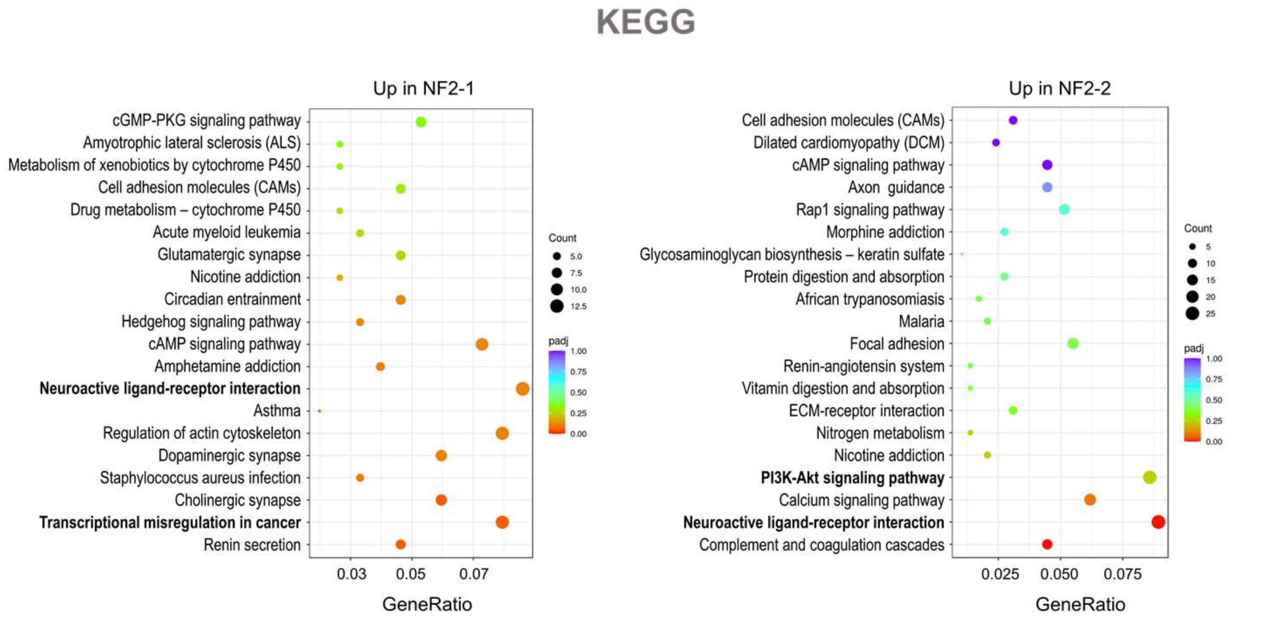

B

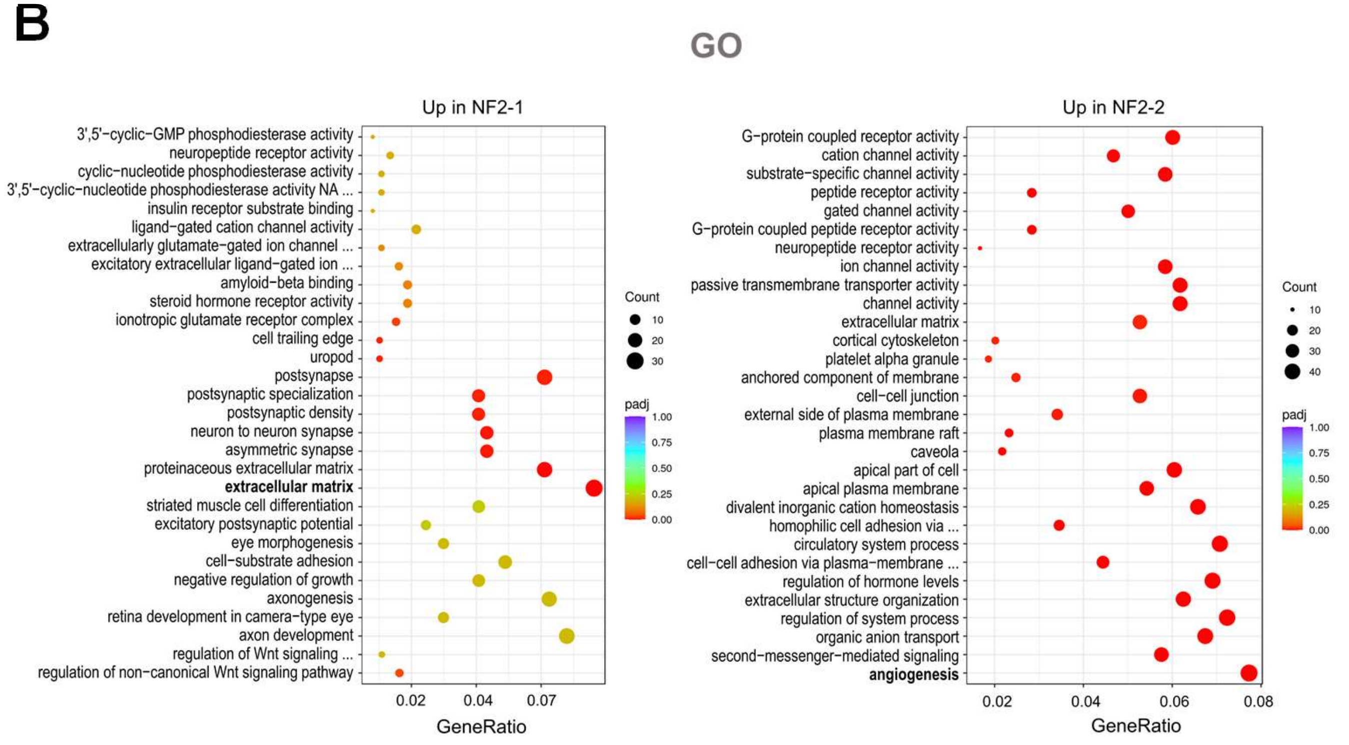

Supplementary Figure 4.

A. Bubble plot of KEGG enrichment analysis of signaling pathways upregulated in NF2-1 (left panel) versus NF2-2 (right panel) meningiomas. Gene ratio (x-axis) is the percentage of significant genes over the total genes in a given pathway. **B.** Bubble plot of GO enrichment analysis of signaling pathways upregulated in NF2-1 (left panel) versus NF2-2 (right panel) meningiomas.

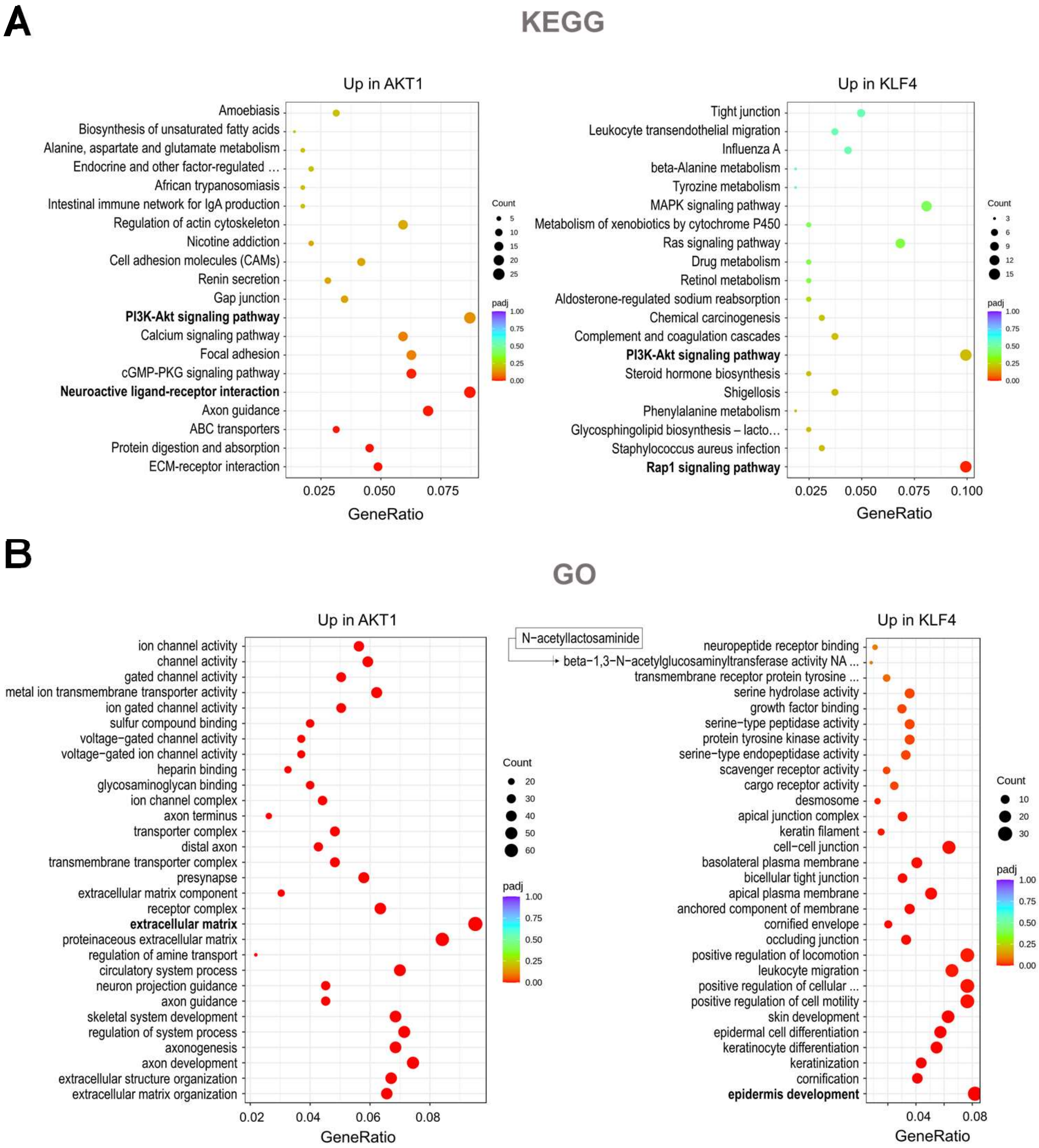

Supplementary Figure 5.
A. Bubble plot of KEGG enrichment analysis of signaling pathways upregulated in AKT1 (left panel) versus KLF4 (right panel) meningiomas. Gene ratio (x-axis) is the percentage of significant genes over the total genes in a given pathway. **B.** Bubble plot of GO enrichment analysis of signaling pathways upregulated in AKT1 (left panel) versus KLF4 (right panel) meningiomas.
